# Toxin–Antitoxin Systems in Pathogenic Bacteria

**DOI:** 10.3390/toxins13020074

**Published:** 2021-01-20

**Authors:** Juan C. Alonso

**Affiliations:** Department of Microbial Biotechnology, Centro Nacional de Biotecnología, CNB-CSIC, 28049 Madrid, Spain; jcalonso@cnb.csic.es

Toxin–antitoxin (TA) systems, which are ubiquitously present in plasmids, bacterial and archaeal genomes, are classified as types I to VI, according to the nature of the antitoxin and to the mode of toxin inhibition [1,2,3,4,5]. TA systems are not essential for normal cell proliferation, but they control a diverse repertoire of cell transition states in response to various environmental stresses [1,2,3,4,5]. In order to survive such stress, cells slow down their growth rate and redirect their metabolic resources until conditions improve and growth can increase [6]. During unperturbed growth, the effect of a type II toxin is neutralized by its binding to a cognate antitoxin, but under certain stress conditions, the unstable antitoxin is rapidly degraded, enabling the stable toxin to reversibly block cell proliferation, without cell lysis [1,2]. The free toxin triggers a dormant state, thus protecting cells from deleterious environments [1,2]. The toxins are bacteriostatic unless neutralized by their cognate antitoxin. Indeed, when the stress is overcome, the levels of the antitoxin rise, the toxin is neutralized and the cell returns to unperturbed growth [1,2,3,4,5]. Toxins are implicated in the fine tuning of multiple cellular processes, such as transcription, translation, DNA replication, the regulation of nucleotide pool, cell-wall synthesis, biofilm formation, phage predation, etc. [1,2,3,4,5].

This Special Issue is focused on understanding the unique response of TAs to stress, the contribution for the maintenance of drug-resistant strains, and their contribution to therapy failure and the development of chronic and recurrent infections. Understanding how TAs contribute to the mechanisms of phenotypic heterogeneity and pathogenesis may enable the rational development of new treatment for infections caused by pathogens. A review paper provides a good overview of how the widespread family of membrane active peptides, Fst/Ldr, is regulated by small RNAs in the TA type I system [7]. Weaver’s review shows that the regulation of the Fst and Ldr toxins is distinct in their respective Gram-positive and Gram-negative hosts, but the effects of ectopic over-expression are similar. Limited toxin expression could conceivably function to slow bacterial growth and halt cell proliferation, playing its canonical role in plasmid stabilization [7].

A manuscript and a review describe specific aspects of the tight control that the TA interaction requires to ensure protection of the cell, and potentially to limit cross-talk between TA pairs of the same family. While each toxin interacts with its cognate antitoxin, TA systems from the same family might present non-cognate interactions and regulate the expression of non-cognate systems. Tandon et al. performed a comprehensive computational analysis on the available 3D structures and generated structural models of paralogous of VapBC and MazEF TA systems [8]. They concluded that for a majority of the systems, the non-cognate TA interactions are structurally incompatible, except for complexes such as VapBC15 and VapBC11, which show similar interfaces and a potential for cross-reactivity. This work contributes to the understanding of TA interfaces and it offers a structure-based explanation for non-cognate toxin–antitoxin interactions [8]. Tu et al. revised the concept that a toxin paralogue may provide a “cure” against the acquisition of highly similar TA systems, such as those found on plasmids or invading genetic elements that frequently carry virulence and resistance genes [9]. Only limited cross-reactions have been observed between chromosomal and mobile genetic elements systems, perhaps due to bias in the type of experiments and functions of TA systems pursued. The Ariyachaokun et al. paper examined the expression of the *mbcAT* operon and its regulation [10]. The *Mycobacterium tuberculosis* type II MbcT toxin halts cell proliferation through the phosphorolysis of the essential metabolite NAD^+^, and its effect is neutralized by physical interaction with its cognate antitoxin MbcA. The authors developed a dual fluorescent reporter system, which was used to dissect the operon promoter/operator region at the genetic level. Using this system, it was demonstrated that transcription from the *PmbcA* promoter is induced by a range of stress conditions, reflecting those encountered inside the infected host and uncovering that this TA system could be exploited to treat tuberculosis [10].

Two papers within this Special Issue are focused on different aspects of toxins of the MazF/PemK superfamily that cleaves mRNA in a sequence-specific and ribosome-independent manner [11,12]. Kang et al. conducted an in-depth structural and functional analysis on the mRNA interferase (RNase) of the MazF/PemK family in *Bacillus cereus* [11]. Bleriot et al. studied the molecular mechanisms associated with chlorhexidine (CHLX) adaptation in two clinical strains of *Klebsiella pneumoniae* by phenotypic and transcriptomic analyses and their association with a new PemK/PemI TA system [12]. Klimkaitè et al. have found by bioinformatic analysis 49 putative TA systems in *Stenotrophomonas maltophilia*, and asked whether clinical and environmental isolates contain a different set of type II TA systems [13]. The authors observed that RelBE, HicAB, and the previously undescribed COG3832-ArsR operon were present solely in clinical *S. maltophilia* isolates collected in Lithuania, while HipBA was more frequent in the environmental ones. The paper by Moreno-Del Alamo et al. explores toxin ζ, which reduces the ATP and GTP levels, increases the (p)ppGpp and c-di-AMP pool and inactivates a fraction of uridine diphosphate-N-acetylglucosamine (UNAG), transiently inducing reversible dormancy. The authors, using a genetic orthogonal control of toxin ζ and antitoxin ε levels in *B. subtilis* cells, have shown that transient toxin ζ expression causes a metabolic heterogeneity that induces toxin and Amp dormancy over a long window of time rather than cell persistence. Antitoxin ε expression, by reversing ζ activities, facilitates the exit of Amp-induced dormancy both in *rec*^+^ and *recA* cells. It has been proposed that an unexploited target to fight against antibiotic persistence is to disrupt toxin–antitoxin interactions [14]. Lastly, the paper by Tuchscherr et al. examined how the TA systems could be developed as targets for novel antimicrobials, and discussed possible undesirable effects of such therapeutic intervention, such as the induction of persister cells, biofilm formation and toxicity in eukaryotic cells [15].
